# Accurate short-read alignment through *r*-index-based pangenome indexing

**DOI:** 10.1101/gr.279858.124

**Published:** 2025-07

**Authors:** Rahul Varki, Massimiliano Rossi, Eddie Ferro, Marco Oliva, Erik Garrison, Ben Langmead, Christina Boucher

**Affiliations:** 1 Department of Computer and Information Science and Engineering, University of Florida, Gainesville, Florida 32611, USA;; 2 Department of Genetics, Genomics, and Informatics, University of Tennessee Health Science Center, Memphis, Tennessee 38163, USA;; 3 Department of Computer Science, John Hopkins University, Baltimore, Maryland 21218, USA

## Abstract

Aligning to a linear reference genome can result in a higher percentage of reads going unmapped or being incorrectly mapped owing to variations not captured by the reference, otherwise known as reference bias. Recently, in efforts to mitigate reference bias, there has been a movement to switch to using pangenomes, a collection of genomes, as the reference. In this paper, we introduce Moni-align, the first short-read pangenome aligner built on the *r*-index, a variation of the classical FM-index that can index collections of genomes in O(*r*)-space, where *r* is the number of runs in the Burrows–Wheeler transform. Moni-align uses a seed-and-extend strategy for aligning reads, utilizing maximal exact matches as seeds, which can be efficiently obtained with the *r*-index. Using both simulated and real short-read data sets, we demonstrate that Moni-align achieves alignment accuracy comparable to vg map and vg giraffe, the leading pangenome aligners. Although currently best suited for aligning to localized pangenomes owing to computational constraints, Moni-align offers a robust foundation for future optimizations that could further broaden its applicability.

Pangenome references enable read aligners to account for intra-species variations. Originally developed from bacterial studies aimed at understanding the complete genetic diversity of bacterial genes, the concept of the pangenome has been expanded to encompass the full genomic variation within any biological population (Golicz et al. 2020; Abondio et al. 2023). Because of lower sequencing costs, building pangenomes for larger organisms has become viable for the first time. In the past decade alone, groups such as All of Us (The All of Us Research Program Investigators 2019), the Human Pangenome Reference Consortium (HPRC) (Wang et al. 2022), the Chinese Pangenome Consortium (Gao et al. 2023), the 1000 Genomes Project (The 1000 Genomes Project Consortium 2015), and the 100K Genome Project (Turnbull et al. 2018) have made a significant effort to sequence a large number of individual human genomes. The theoretical and practical challenges now lie in designing read aligners that can efficiently index and align to these larger pangenomes, all while leveraging the invaluable insights they offer.

Before the concept of the pangenome, standard read aligners such as BWA (Li and Durbin 2009; Li 2013) and Bowtie 2 (Langmead and Salzberg 2012) used the full-text index in minute space (FM-index) to index the reference genome. The FM-index is a compressed, lossless, text-based index consisting of the Burrows–Wheeler transform (BWT) (Burrows and Wheeler 1994), the suffix array (SA) (Manber and Myers 1993), and small auxiliary data structures to support rank queries on the BWT. The index supports fast locate and count queries, making it ideal for a read aligner index. The downside of the FM-index is its linear space requirement relative to the length of the uncompressed text, which poses a major restriction for indexing pangenome references. As a result, there has been growing interest in developing aligners specifically designed to handle pangenome references. Consequently, two pangenomic aligners have been developed within the vg toolkit: vg map (Garrison et al. 2018; Sirén et al. 2020) and vg giraffe (Sirén et al. 2021). Both methods involve constructing and aligning to a variation graph. In this graph, nodes represent segments of DNA sequences, which can be shared across multiple genomes or can be unique to individual ones, and edges indicate the relationships between these sequences, showing how segments are connected in different genomes. By walking through the graph, it is possible to reconstruct individual genome sequences or identify common and divergent regions across multiple genomes. Common regions in the pangenome are collapsed into single nodes and single edges, making the representation efficient for pangenomes with a lot of shared DNA. For collections of highly repetitive genomes, graphs can efficiently compress the pangenome. However, there are downsides to graph-based pangenome aligners. Graphs can quickly become complex as the size of the pangenome grows. When graphs become too complex, a common method is to prune the graph, but it is often unclear what gets removed. For example, pangenome graphs struggle to represent highly repetitive regions like alpha satellites, and these regions are thus commonly removed. This process can also inadvertently remove other important regions, such as protein-coding genes and inversion polymorphisms (Liao et al. 2023; Porubsky et al. 2023). It is also possible to create chimeric sequences in the graph that consist of known alleles but in combinations not observed in nature, which can result in alignments to haplotypes that do not actually exist in the pangenome (Sirén et al. 2020). A current challenge in the field is the lack of a viable alternative to graph-based pangenome aligners.

The *r*-index has emerged as a promising alternative to graph-based indexes, demonstrating scalability with the expanding size of the pangenome. It is a compressed variation of the FM-index that harnesses the repetitive nature of the pangenome. The size of the *r*-index is determined by the number of maximal same-character runs in the BWT, which is denoted as *r*. This is because the *r*-index stores only a single entry of the BWT and the SA for each of the *r* runs in order to support the same queries as the FM-index (Gagie et al. 2018). A small auxiliary data structure is then used to efficiently identify any missing entries. The details of the *r*-index are discussed in the Methods. In practice, the number runs in the BWT grows sublinearly, as repetitive regions of the pangenome only extend existing runs rather than increase their number (Boucher et al. 2018; Kuhnle et al. 2020). The *r*-index addresses the space complexity of the FM-index by leveraging the pangenome's repetitiveness, similar to graph-based approaches, while retaining the FM-index's desirable lossless, text-based properties. Although a complete construction algorithm for the *r*-index has been developed recently (Rossi et al. 2022), a corresponding read aligner based on this index has yet to be fully implemented.

Here, we introduce Moni-align, the first pangenomic short-read (100 bp–250 bp) aligner that is based on the *r*-index. Moni-align aligns reads to the pangenome using a seed-and-extend strategy using maximal exact matches (MEMs) as seeds, a proven read-alignment strategy (Li 2013, 2018). This is possible because of recent work showing that the *r*-index, when augmented with a small auxiliary thresholds’ data structure, can support finding MEMs efficiently (Rossi et al. 2022). Moni-align initially aligns to the genome(s) in the pangenome but lifts the alignments back to the linear reference genome, enabling its alignment SAM file to be compatible with existing downstream analysis software. Through our experiments, we demonstrate that Moni-align is an effective tool for accurate pangenome read alignment and is adept at handling complex genomic variation and integrating with existing analysis pipelines. Although the current version is most effective in aligning to more localized pangenomes owing to computational resource demands, this presents an opportunity for future optimizations to further broaden its applicability.

## Results

### Performance evaluation on MHC region

#### Experimental setup

In this experiment, we evaluated Moni-align's alignment accuracy within the major histocompatibility complex (MHC) region. To do this, we aligned five subsets of HG005 Chr 6 reads (2 × 250 bp) with Moni-align (v1.0.0), vg map (v1.49.0) (Garrison et al. 2018; Sirén et al. 2020), and vg giraffe (v1.49.0) (Sirén et al. 2021) to both the GRCh38 Chr 6 linear reference and a Chr 6 pangenome reference containing HPRC variants restricted to the MHC region (Chr 6: 28,510,128–33,480,000), excluding nonreference alleles outside this region. For each tool, we built the pangenome using the GRCh38 Chr 6 FASTA file (https://www.ncbi.nlm.nih.gov/datasets/genome/GCF_000001405.40) and a variant call format (VCF) file of the MHC region, which we constructed using the maternal and paternal haplotype-resolved assemblies of the 47 individuals included in the HPRC year 1, version 2 data freeze (https://human-pangenomics.s3.amazonaws.com/index.html) in addition to T2T-CHM13 (https://github.com/marbl/CHM13) and GRCh38. To construct the pan-MHC VCF file, we first performed an all-to-all alignment of the input assemblies using wfmash (v0.13.0) (https://zenodo.org/doi/10.5281/zenodo.10864529), extracted assembly regions corresponding to the GRCh38 MHC region using impg (v0.2.0) (https://github.com/pangenome/impg), built a pangenome graph from these regions with pggb (v0.6.0) (Garrison et al. 2024), and finally called variants with the graph using vg (v1.55.0) (Garrison et al. 2018; Sirén et al. 2020). An odgi (v0.8.6) (Guarracino et al. 2022) visualization of the region is provided in Supplemental Figure S1. We provide further construction details in the Supplemental Experiment Methods. The reads were extracted from the HG005 Genome in a Bottle (GIAB) 300× 2 × 250 bp Illumina Novoalign BAM file, specifically those aligned to Chr 6 (https://github.com/genome-in-a-bottle/giab_data_indexes). We downsampled the extracted Chr 6 read set into five subsets, each with 30×–35× coverage, containing 11,998,425 paired-end reads (23,996,850 total reads) on average. We specifically chose to align reads from HG005 owing to this sample being the son in the well-known Han Chinese trio, and both its maternal and paternal haplotype-resolved assemblies were used in the VCF construction. Our analysis focused on the read alignments to the MHC region owing to its polymorphic nature (Gaudieri et al. 2000; Janeway et al. 2001; Garrigan and Hedrick 2003; Piertney and Oliver 2006), in which the increased genetic diversity captured by the pangenome would likely enable more accurate alignments.

We built the indexes of the linear and pangenome references used in this experiment with the default indexing commands of Moni-align, vg map, and vg giraffe. We used the default alignment commands for both vg map and vg giraffe. For Moni-align, we set the minimum MEM length to 25 bp (−l 25), retained up to 1000 occurrences of each MEM per genome (−S 1000), and disabled the MEM frequency (−f), the MEM orientation (−d), and the chain (−a) filters, in addition to disabling the orphan recovery (−u) feature. Details on each setting for Moni-align can be found in the Methods. The indexing and alignment commands were run with 32 threads; however, we report CPU time in the paper. The full indexing and alignment commands can be found in the Supplemental Experiment Methods.

The experiment was conducted with Snakemake (v7.32.4) (Köster and Rahmann 2012) on a server with 512 GB of RAM and a 3.35 GHz AMD EPYC 7702 64-core processor, running Red Hat Enterprise Linux 8.8. The compiler used was g++ version 9.3.0. The time and memory values reported were obtained from the Snakemake benchmark files.

#### Alignment to MHC pangenome

Moni-align balanced indexing speed and memory efficiency, delivering moderate yet competitive performance compared with vg map and vg giraffe, although its alignment resource usage still offered room for improvement. The indexing and alignment benchmarks for both the linear and pangenome references are provided in Tables 1 and 2, respectively, for each aligner. The tables show that Moni-align ranked between vg map and vg giraffe in CPU time and peak memory usage when indexing the pangenome, using more resources than vg giraffe but fewer than vg map. Moni-align required more CPU time and peak memory for the pangenome alignments compared with vg map and vg giraffe, running at least an order of magnitude slower and using three to five times more peak memory. The bottleneck in Moni-align's alignment resource usage primarily arises from its current method of finding and storing MEM occurrences between the reads and reference. Although the peak memory usage of Moni-align can be partially controlled by the user through the various MEM filters, these filters are mainly after processing, meaning that Moni-align must first spend time retrieving all the valid MEMs before retaining only a portion of them, even when the filters are applied.

**Table 1.** Statistics for indexing and aligning to GRCh38 Chr 6

Aligner	Moni-align	vg map	vg giraffe
Total Chr 6 reads	23,996,850 ± 6941	23,996,850 ± 6941	23,996,850 ± 6941
Chr 6 aligned reads	21,735,403 ± 5668	23,830,814 ± 6628	23,996,324 ± 6927
MHC aligned reads	579,417 ± 1032	639,421 ± 774	644,479 ± 761
Index CPU time (min)	56	129	4
Index peak memory (GB)	3.93	14.31	4.69
Align CPU time (min)	1102 ± 281	795 ± 73	81 ± 17
Align peak memory (GB)	2.55 ± 0.01	2.22 ± 0.06	4.02 ± 0.01

Average and standard deviation for each category, calculated from the alignments of the five HG005 read subsamples produced by the three aligners.

**Table 2.** Statistics for indexing and aligning to the pan-MHC Chr 6 pangenome

Aligner	Moni-align	vg map	vg giraffe
Total Chr 6 reads	23,996,850 ± 6941	23,996,850 ± 6941	23,996,850 ± 6941
Chr 6 aligned reads	21,864,028 ± 6234	23,831,015 ± 6605	23,996,303 ± 6931
MHC aligned reads	584,793 ± 1059	640,116 ± 751	644,751 ± 746
Index CPU time (min)	107	109	4
Index peak memory (GB)	7.36	14.68	4.85
Align CPU time (min)	16,486 ± 1936	894 ± 376	258 ± 382
Align peak memory (GB)	11.48 ± 0.73	2.25 ± 0.17	4.05 ± 0.01

Average and standard deviation for each category, calculated from the alignments of the five HG005 read subsamples produced by the three aligners.

We found that Moni-align could achieve alignment rates comparable to that of vg map and vg giraffe. As shown in Tables 1 and 2, Moni-align with its orphan recovery feature disabled had an average alignment rate of 91% when aligning the Chr 6 reads to the pangenome. The majority of the 2,132,822 reads on average that Moni-align did not align were orphaned reads, paired-end reads in which only one read mapped to the reference. We separately tested whether Moni-align could recover these reads using its orphan recovery feature and found that it could successfully recover about 1.48 million of these reads on average, resulting in total alignment rates of 97%–98%, only slightly lower than the average 99% alignment rate of both vg map and vg giraffe. We theorize that the reason Moni-align could not recover the remaining reads is that key MEMs between the read and the reference were smaller than the minimum MEM threshold value set. Despite its utility, we chose to disable Moni-align's orphan recovery feature in this experiment because it significantly increased the CPU time, nearly doubling it.

**Figure 1.**
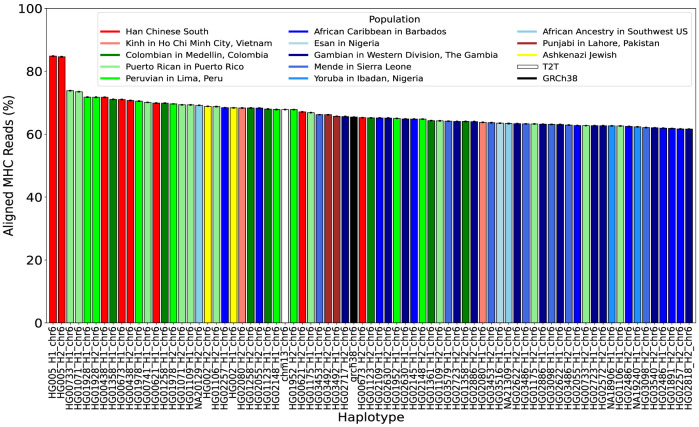
Top 75 haplotype matches for HG005 MHC-aligned reads. Each bar shows the percentage of HG005 MHC-aligned reads that best align to a specific haplotype in the pan-MHC Chr 6 pangenome, using alignments determined by Moni-align. The percentage reflects an average across five read subsamples, with error bars indicating the standard deviation. Bar colors broadly represent the population origin of each haplotype: red for Southeast Asian, green for South American, and blue for African ancestry. Additionally, the black bar represents GRCh38, and the white bar represents T2T-CHM13.

Based on the alignments, we found strong evidence that Moni-align accurately identified the correct haplotypes for alignment in the pangenome. We analyzed which haplotypes in the pangenome yielded the highest scoring alignments for the MHC-aligned reads across the five alignments generated by Moni-align. Figure 1 shows the top 75 haplotypes with the highest-scoring alignments for the largest percentage of MHC-aligned reads, sorted in descending order. When aligning to a collection of intra-species genomes, a read can often align best to multiple genomes, which is why the percentages in Figure 1 do not sum to 100%. As expected, Figure 1 reveals that Moni-align identified the majority of the MHC-aligned reads as best aligning to HG005's maternal and paternal haplotypes. On average, 85% of these reads best aligned to these haplotypes, with the next closest haplotype having a best alignment rate of 74%, more than 11 percentage points lower. This finding highlights the genetic diversity of individuals, especially in polymorphic regions like the MHC region. In contrast, only an average of 67% and 65% of the MHC-aligned reads aligned best to the T2T-CHM13 and GRCh38 reference, respectively, underscoring the advantage of aligning to a pangenome rather than a consensus linear reference. Additionally, Figure 1 reveals a clear division between the haplotypes positioned to the left and right of the GRCh38 reference. The haplotypes on the left are primarily of Southeast Asian and South American ancestry, whereas those to the right are predominantly of African ancestry. This finding aligns with our expectations, as HG005 is of Southeast Asian ancestry, and there is documented history of Asian migration to South America (Hu-DeHart 2021; Palma 2024) but less to Africa. We could not verify whether these findings hold with the vg map and vg giraffe alignments because these methods do not strictly align to the input haplotypes and thus do not make this information available.

**Figure 2.**
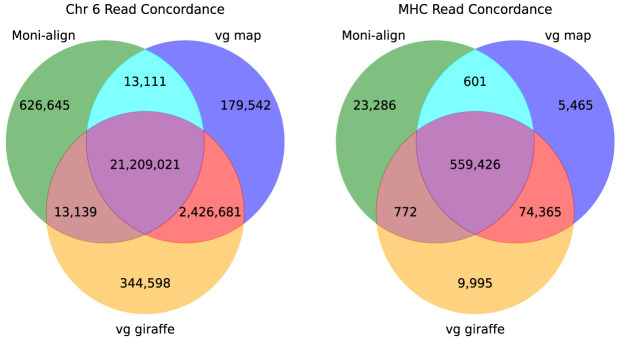
Chr 6 and MHC read concordance. Venn diagrams showing the read concordance of a representative subsample of HG005 Chr 6 reads aligned by Moni-align, vg map, and vg giraffe to the pan-MHC Chr 6 pangenome. The diagram on the *left* shows the read concordance of the Chr 6 aligned reads, and the *right* diagram shows the read concordance of the MHC aligned reads. A read is considered concordant if its mapping position by one aligner is within 10 bp of another.

Assessing the accuracy of read alignments with real data is challenging because of the lack of a ground truth. To compensate for the lack of ground truth, we calculated the alignment concordance between the read alignments of Moni-align, vg map, and vg giraffe to Chr 6 and its MHC region using a representative pangenome aligned subsample as shown in Figure 2. In this figure, a read is considered concordant if the mapping position reported by one aligner is within 10 bp of another aligner. We observed high concordance among the three aligners for the Chr 6 aligned reads, with an 88% concordance rate. Concordance rates increased to 93% when comparing with the Moni-align alignments generated with the orphan recovery feature, resulting in the concordance rate between only vg map and vg giraffe to decrease. Relaxing the threshold from 10 bp to 250 bp (the read length) increased the concordance rates from 88% to 91% and from 93% to 96% when comparing the Moni-align alignments generated without and with orphan recovery, respectively. The observed increase is because of a portion of the reads mapping to tandem repeat regions >10 bp. We could not calculate concordance rates for only the MHC-aligned reads because the true number of reads that should be mapped to this region is unknown. Although the alignments were highly concordant, this did not necessarily reflect their quality. We indirectly assessed the quality of the alignments by calling variants and comparing them to a ground-truth set.

#### Improvement of MHC variant calling with pangenome alignments

We called HG005 variants from the MHC region with DeepVariant (v1.5.0) (Poplin et al. 2018) using the alignments from both the linear and pangenome reference. We compared the called HG005 MHC variants against the ground-truth GIAB HG005 MHC variants (https://ftp-trace.ncbi.nlm.nih.gov/ReferenceSamples/giab/release/ChineseTrio/HG005_NA24631_son/NISTv4.2.1/GRCh38/) with Illumina's hap.py script (v0.3.15) (Krusche et al. 2019) and RTG's vcfeval (v3.12.1) (Cleary et al. 2015) as the comparison engine. The hap.py script benchmarks variant calls at the haplotype level and, combined with vcfeval, provides a robust method for resolving differences in variant representations. The analysis was restricted to high-confidence MHC regions defined in the GIAB HG005 BED file (https://ftp-trace.ncbi.nlm.nih.gov/ReferenceSamples/giab/release/ChineseTrio/HG005_NA24631_son/NISTv4.2.1/GRCh38/).

**Figure 3.**
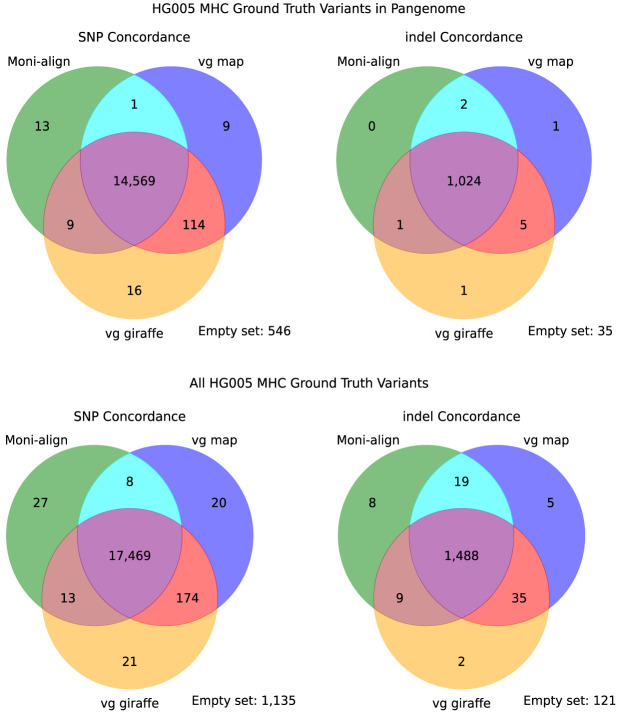
SNP and indel variant concordance. Venn diagrams illustrating the concordance of SNPs and indels called by DeepVariant from a representative subsample of HG005 Chr 6 reads aligned by Moni-align, vg map, and vg giraffe to the pan-MHC Chr 6 pangenome. The *top* row shows the concordance of GIAB HG005 MHC variants present in the pangenome, restricted to the high-confidence MHC regions, identified by DeepVariant. The *bottom* row shows the concordance of the full set of GIAB HG005 MHC variants, similarly restricted to high-confidence MHC regions, identified by DeepVariant. The number at the *bottom right* of each diagram indicates the number of variants that were not identified by any of the three aligners.

Across all three aligners, aligning to the pangenome helped DeepVariant call novel variants absent from the pangenome, as shown in Figure 3. The full set of GIAB HG005 variants in the high-confidence MHC regions included 18,867 SNPs and 1687 indels, of which 15,277 (81.0%) SNPs and 1069 (63.4%) indels were present in the HPRC pan-MHC VCF file. We confirmed that DeepVariant called most of the high-confidence GIAB HG005 variants present in the HPRC pan-MHC VCF file from the pangenome alignments of all three aligners. From Moni-align, DeepVariant called 14,592 (95.5%) SNPs and 1027 (96.1%) indels found in the pangenome. For vg map, DeepVariant called 14,693 (96.1%) SNPs and 1032 (96.5%) indels. For vg giraffe, DeepVariant called 14,708 (96.3%) SNPs and 1031 (96.4%) indels. Compared with the full set of GIAB HG005 MHC variants, the number of shared variants increased. For example, DeepVariant called 17,517 (92.8%) SNPs and 1524 (90.3%) indels from the Moni-align alignments that were part of the full set. With vg map, DeepVariant called 17,671 (93.7%) SNPs and 1547 (91.7%) indels. With vg giraffe, DeepVariant called 17,677 (93.7%) SNPs and 1534 (90.9%) indels. These results demonstrate that Moni-align, along with vg map and vg giraffe, can generate alignments that support the discovery of novel variants absent from the pangenome.

**Figure 4.**
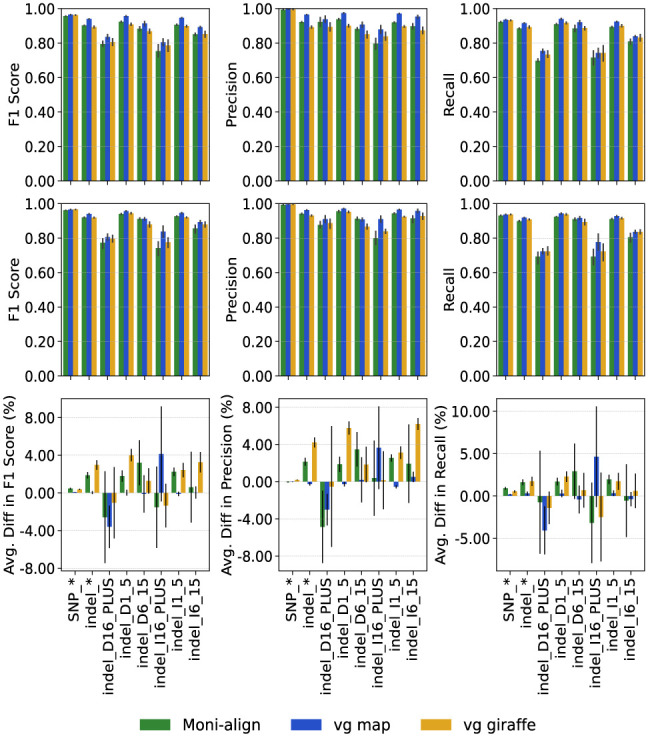
MHC variant comparison of the pangenome aligners. The *top* row, from *left* to *right*, displays bar plots of the F1, precision, and recall scores for the SNPs and indels called by DeepVariant from the linear MHC reference alignments. The *middle* row displays the same metrics for the pan-MHC reference alignments. The *bottom* row, from *left* to *right*, displays the average percentage differences for the F1, precision, and recall metrics between the pan-MHC and linear MHC reference alignments. The aligners are indicated by the color of the bar with green corresponding to Moni-align, blue corresponding to vg map, and orange corresponding to vg giraffe. The error bars are the standard deviations. Note that the *x*-axis categories have a type subtype notation. The two types are SNP and indel. Indels are further categorized as insertions (I) and deletions (D). The subtypes are as follows: (asterisk) all subtypes, (1_5) 1–5 bp, (6_15) 6–15 bp, and (16_PLUS) ≥16 bp.

With all three aligners, aligning to the pangenome reference improved DeepVariant's F1-scores for SNPs and indels in the MHC region compared with the linear reference. Figure 4 shows the average F1-score, precision, and recall for DeepVariant-called variants in the high-confidence MHC regions, using the linear and pangenome alignments of Moni-align, vg map, and vg giraffe, along with their percentage differences across different variant types. With Moni-align, the average F1-score increased by 0.45% (±0.09%) for SNPs and 1.87% (±0.34%) for indels. vg giraffe showed similar improvements, with an average F1-score increase of 0.37% (±0.07%) for SNPs and 2.96% (±0.50%) for indels. In contrast, vg map's pangenome alignments showed minimal improvement, with average F1-score increases of only 0.08% (±0.02%) for SNPs and 0.02% (±0.20%) for indels. However, the precision and recall plots reveal that vg map's pangenome alignments enabled DeepVariant to correctly call more large insertion variants of ≥16 bp than its linear reference alignments and, thus, did benefit from aligning to the pangenome.

Overall, despite the varying F1-score increases, all three aligners achieved similar DeepVariant F1-scores for called SNPs and indels in the MHC region using the pangenome reference. For SNPs, vg giraffe had the highest average F1-score at 0.965, just 0.004 higher than Moni-align, which had the lowest average F1-score at 0.961. For indels, vg map had the highest average F1-score at 0.940, only 0.021 higher than vg giraffe, which had the lowest average F1-score at 0.919. These similar high F1-scores for both SNPs and indels observed across all three aligners correlate with the high read concordance rates observed earlier, confirming the alignments were of high quality.

### Performance evaluation on simulated data

#### Experimental setup

In this experiment, we benchmarked Moni-align against leading pangenome and standard aligners on simulated data, assessing its performance across key metrics relevant for pangenome aligners. We simulated 1 million 2 × 100 bp paired-end reads (2 million total reads) from Chr 21 of HG002 using Mason2 with its default Illumina settings (Holtgrewe 2010). These reads were then aligned to both the GRCh38 Chr 21 reference and increasingly larger Chr 21 pangenomes, containing variants ranging from 10 to 500 haplotypes from the 1000 Genomes Project (http://ftp.ensembl.org/pub/data_files/homo_sapiens/GRCh38/variation_genotype/ALL.chr21_GRCh38.genotypes.20170504.vcf.gz) (The 1000 Genomes Project Consortium 2015). The larger pangenomes included all variants from the smaller pangenomes. Additional details about the pangenomes used in this experiment can be found in the Supplemental Experiment Methods. We ensured that HG002 was excluded from the samples selected for the pangenomes. Our evaluation focused on the scalability of Moni-align (v1.0.0) compared with other graph-based pangenome aligners such as vg map (v1.49.0) (Garrison et al. 2018; Sirén et al. 2020), vg giraffe (v1.49.0) (Sirén et al. 2021), HISAT2 (v2.2.1) (Kim et al. 2019), GraphAligner (commit 4c44e5d) (Rautiainen and Marschall 2020), and standard aligners including Bowtie 2 (v2.4.5) (Langmead and Salzberg 2012), BWA (v0.7.17) (Li and Durbin 2009; Li 2013), and minimap2 (v2.2.4) (Li 2018). We assessed several key metrics, such as index construction CPU time and peak memory usage, alignment CPU time and peak memory usage, alignment precision and recall, and mapping quality assignment.

We built the indexes of the linear and pangenome references used in this experiment with the default indexing commands of Moni-align, vg map, vg giraffe, HISAT2, GraphAligner, Bowtie 2, BWA, and minimap2. For Moni-align's alignment settings, we set the minimum MEM length to 25 bp (−l 25), retained up to 2000 occurrences of each MEM per genome (−S 2000), disabled the MEM frequency (−f) and orientation filters (−d), and kept the chain filter and orphan recovery feature enabled. For the other aligners, we primarily used their default alignment settings. Details on each setting for Moni-align can be found in the Methods. The indexing and alignment commands were run with 32 threads when possible; however, we report CPU time in the paper to give a fair comparison. All indexing and alignment commands can be found in the Supplemental Experiment Methods.

The experiment was conducted with Snakemake (v7.32.4) (Köster and Rahmann 2012) on a server with 512 GB of RAM and a 3.35 GHz AMD EPYC 7702 64-Core Processor, running Red Hat Enterprise Linux 8.8. The compiler used was g++ version 9.3.0. The time and memory values reported were obtained from the Snakemake benchmark files.

#### Evaluation of the index construction scalability

**Figure 5.**
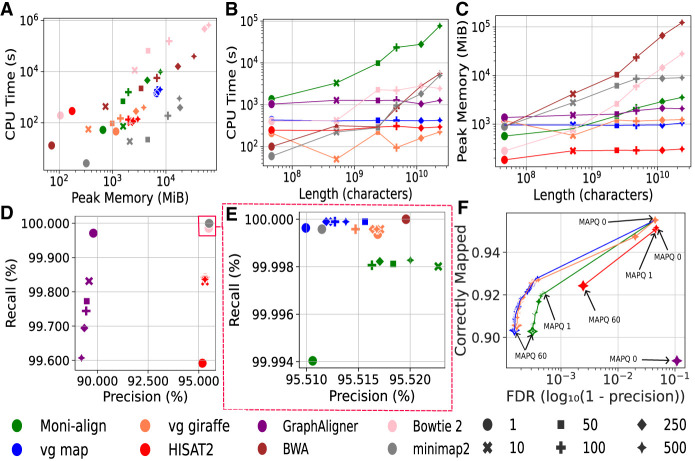
Chr 21 pangenome experiment. The subfigures compare different performance metrics across all aligners. Marker color indicates the aligner, and marker shape indicates the number of haplotypes in the pangenome reference. (*A*) CPU time and peak memory usage for building the Chr 21 pangenome indexes for all aligners, excluding GraphAligner owing to its lack of an in-built indexing method. (*B*) CPU alignment times. (*C*) Peak memory usage during alignments. (*D*) Precision and recall of the alignments, excluding the pangenome alignments of Bowtie 2, BWA, and minimap2 owing to their lack of liftover functionality. Most markers are within the red rectangle. (*E*) Precision and recall of alignments located within the red rectangle in *D*. (*F*) The mapping quality curves of the alignments produced by the pangenome aligners to the 500-haplotype Chr 21 pangenome.

Figure 5A compares the indexing CPU time and peak memory usage of Moni-align with the other aligners. GraphAligner was not included in our evaluation owing to its lack of an in-built indexing method. We found that Moni-align indexed the Chr 21 pangenomes significantly faster and with less memory than both BWA and Bowtie 2, and the advantage grew to be orders of magnitude for the larger pangenomes. This result was expected, as it further confirmed the theory that the *r*-index construction scales better than that of the FM-index for indexing repetitive pangenomes.

Among all the aligners, Moni-align's indexing resource usage was positioned between that of BWA and Bowtie 2 (FM-index based) and that of vg map, vg giraffe, and HISAT2 (graph-based) for most of the pangenomes indexed. We note that although Moni-align's indexing time and memory usage is significantly better than that of building the FM-index, these resources are still tied to the total length of the pangenome owing to the *r*-index being a lossless text-based index. In contrast, the indexing time and memory usage of vg map, vg giraffe, and HISAT2 depend more on the size of the VCF file representing the multiple aligned sequences. This explains the differing resource usage trends between the tools. Nonetheless, Moni-align's index construction method remains directly competitive with the graph-based pangenome aligners for indexing large pangenomes.

#### Evaluation of the alignment scalability

The CPU time and peak memory usage for aligning the reads to the Chr 21 pangenome indexes across all aligners are shown in Figure 5, B and C. We see from the figures that Moni-align did the alignments with comparable peak memory but was slower than the graph-based aligners. The alignment slowdown occurs because Moni-align currently needs to process the full set of valid MEMs across the pangenome before the alignment, and extracting the full set of MEMs can be time-consuming depending on the quantity. This time can be reduced in the future with further improvements to the index that allow us to have greater control over the MEM finding queries. Moni-align's memory footprint is more comparable to the graph-based aligners owing to its ability to filter MEMs likely to be uninformative. From Figure 5C, we see that the memory usage of Moni-align and the other graph-based aligners is orders of magnitude smaller than that of the standard aligners when aligning to the larger pangenomes. It is worth noting that for pangenome alignment, memory requirements are often a more limiting factor than alignment time.

#### Evaluation of the pangenome alignment accuracy

We calculated the precision and recall of the alignments to the Chr 21 pangenomes across all aligners, and the results are shown in Figure 5D. We defined true positive (TP), false positive (FP), and false negative (FN) as follows: a TP read is a read mapped within 10 bp of its ground-truth position, an FP read is a read mapped >10 bp away from its ground-truth position, and an FN read is an unmapped read. True-negative (TN) reads did not occur in this context. We adjusted the positions of soft-clipped alignments in this analysis. Because Bowtie 2, BWA, and minimap2 do not offer critical liftover functionality, we could not calculate the precision and recall scores for their pangenome alignments, which were based on ground-truth positions relative to the GRCh38 Chr 21 reference.

Figure 5D clearly shows that Moni-align's pangenome alignments significantly outperformed the alignments of GraphAligner and HISAT2 in both precision and recall. Moni-align achieved high scores for both metrics, indicated by its markers being located within the upper right corner of the plot encompassed within the red rectangle, indicating robust performance across all alignments. In contrast, GraphAligner showed decreasing performance as the pangenome size increased, evidenced by declining precision and recall. Although HISAT2 exhibited a modest increase in recall and slight improvement in precision, it did not reach the levels achieved by Moni-align and the other aligners within the red rectangle in Figure 5D. However, it is important to consider that GraphAligner was primarily designed for long-read alignments and HISAT2 was primarily designed for RNA alignments, which may account for their relatively poor performance with short-read DNA alignments to the pangenome.

Figure 5E shows the precision and recall of the alignments within the red rectangle shown in Figure 5D. Among the tested pangenome aligners, only Moni-align, vg map, and vg giraffe had all their markers located in this region. Moni-align achieved >99.99% recall and >95.51% precision across all alignments to the pangenomes. The differences in precision and recall among these aligners were minimal, with variations in both metrics being <0.1%. Despite the small differences, we observed that Moni-align achieved the highest precision alignments in this experiment. In some pangenome alignments, Moni-align's precision even surpassed that of BWA's linear reference alignment, the best alignment in terms of overall precision and recall in this experiment. We note that the best pangenome alignments were not always to the references with the highest number of haplotypes. A potential reason for this could be that larger reference pangenomes offer more mapping locations, increasing ambiguity and making it harder for the aligner to identify the correct alignment. This observation has been made in previous studies such as the FORGe study (Pritt et al. 2018).

#### Pangenome mapping quality assignment

We analyzed the mapping qualities assigned to the reads mapped to the 500-haplotype Chr 21 pangenome by the pangenome aligners, and the results are shown in Figure 5F. We excluded the mapping qualities of Bowtie 2, BWA, and minimap2 because most were very low, resulting from multiple identical alignments across different haplotypes. We see from the figure that Moni-align's mapping quality curve closely resembles that of vg map and vg giraffe. However, Moni-align stands out by assigning a slightly higher percentage of correctly mapped reads a mapping quality of zero instead of an intermediate value between one and 60, indicating it is more conservative in assigning mapping qualities compared with both vg map and vg giraffe. Importantly, Moni-align, vg map, and vg giraffe were the only aligners to produce the full spectrum of mapping qualities (zero to 60). In contrast, HISAT2 provided only a limited set (zero, one, 60), and GraphAligner assigned only a single mapping quality (zero). Mapping quality plays a critical role in the functionality of downstream analysis software, such as variant callers. Therefore, Moni-align's ability to produce accurate mapping quality estimates for pangenome alignments is a key strength of our aligner.

## Discussion

In this paper, we describe Moni-align, the first short-read pangenome aligner based on the *r*-index. The *r*-index has advantages over graph-based indexes because it can be constructed without a costly multiple alignment step and fully preserves the sequences of all indexed genomes. Moni-align employs a seed-and-extend strategy to align reads to the pangenome using MEMs as seeds. It can properly account for identical alignments across multiple haplotypes through the use of liftover. The Moni-align alignment file is compatible with existing downstream analysis software such as variant callers.

We demonstrate with both simulated and real short-read data sets that Moni-align can produce pangenome alignments that are directly comparable to vg map and vg giraffe, the two currently leading pangenome aligners. This is evidenced by metrics such as alignment precision and recall, mapping quality assignment, and variant calling accuracy. We also show that aligning to a pangenome offers clear benefits over a consensus linear reference, particularly in the MHC variant-calling experiment. In that experiment, aligning to the pangenome improved the F1-scores in calling both SNPs and indels in the MHC region. However, we mention that the reported variant-calling results were likely on the lower end of achievable performance, as the initial read selection was biased to include only previously aligned reads to the GRCh38 Chr 6 reference. Although each subsampled Chr 6 read set averaged 30×–35× coverage, some areas in the MHC region lacked depth in the initial alignment, presumably because of limitations of aligning to the GRCh38 reference. Our approach to read selection prevented Moni-align, vg map, and vg giraffe from attempting to align reads that the initial alignment missed, potentially reducing the number of truth variants that could have been found with the pangenome if we aligned the full read set. We performed the experiment this way because aligning the full-genome read set to a localized pangenome would lead to many FP alignments, and aligning to a full pangenome across all chromosomes would be too computationally expensive with the current version of Moni-align.

We point out that because this is the first-of-its-kind read aligner, it opens the door for future improvements. For example, improving the *r*-index to support more specific MEM queries, such as selecting a reference in the pangenome to retrieve MEM occurrences, could significantly reduce the alignment time. Additionally, improving the base-level alignment time by using the wavefront alignment algorithm (WFA) (Marco-Sola et al. 2021, 2023) warrants future investigation. WFA finds the optimal alignment with runtime proportional to the sequence length and the alignment score, instead of the Needleman–Wunsch algorithm, which has a quadratic runtime. Additionally, in theory, speeding-up the chaining step by vectorizing the chaining of predecessor anchors as proposed by Kalikar et al. (2022) maybe possible in Moni-align because our chaining algorithm resembles that of minimap2.

Lastly, we conclude by noting that the Moni-align framework could be extended to support long-read alignment. The main challenge would be in effectively managing situations in which spurious MEMs are formed or in which MEMs are missing owing to the relatively high error rates of long-read sequences. Future work could explore applying the concept of syncmers (Dutta et al. 2022) or strobemers (Sahlin 2021, 2022) to the context of Moni-align. With further refinement, Moni-align has the potential to enhance both short-read and long-read sequencing applications, broadening the scope of genomic research.

## Methods

This section provides more details on the different steps performed in both the index (moni build) and alignment (moni align) workflows in Moni-align. Both the indexing and alignment steps support multithreading. We begin by giving some background information on some of the algorithmic contributions implemented in Moni-align.

### Preliminaries

#### The r-index

**Figure 6.**
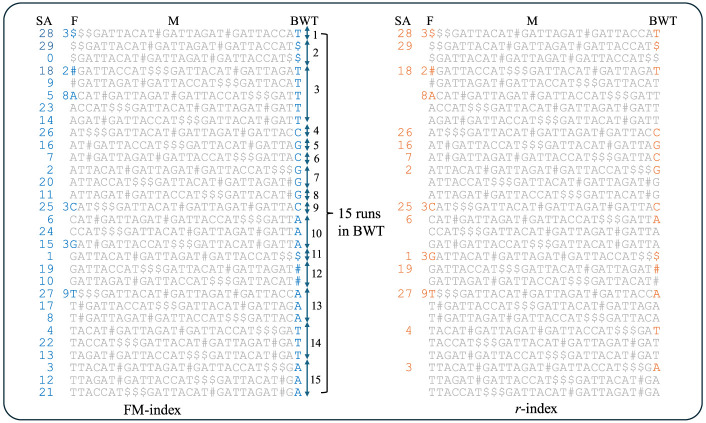
Illustration of the difference between the FM-index and *r*-index. There are 15 runs in the BWT, in which a run is the consecutive occurrence of a single character. The complete BWT matrix consisting of all rotations of the input in lexicographical order. The FM-index consists of SA, BWT array, and the first column of the BWT matrix (F). We note that F can be stored as an integer array consisting of the number of times each character occurs in the input. This compression is illustrated. The *r*-index stores only a single entry of the BWT and SA for each run in the BWT.

The FM-index is the primary data structure behind existing read aligners, which consists of the BWT of the input text, a rank data structure over the BWT, and the SA sampled at constant size intervals (Ferragina and Manzini 2005). Given an input sequence *S*, the BWT considers all possible rotations of *S* sorted in lexicographical order, creating the BWT matrix, which we denote as M. The first column of M (denoted as F) and the last column of M (denoted as L or the BWT) are needed to efficiently support all queries of the form: Given a query sequence *P*, find all occurrences of *P* in *S* (which is referred to as count queries). To locate all these occurrences, the starting location in *S* of each rotation in M is needed, which is the SA. We define a *run* in the BWT as a maximal same-character substring, and denote the number of runs as *r*. If M is constructed on large, repetitive sequences, then *r* is significantly smaller than *n*. Gagie et al. (2018) showed that only the first (or last) entry of the BWT and SA of each run is needed to support count and locate queries. A small auxiliary data structure called φ is used to compute the missing SA values. This data structure—namely, SA sample, BWT, and φ—is referred to as *r*-index and can be stored in O(*r*)-space. This is illustrated in Figure 6.

#### Thresholds

The *r*-index (and FM-index) cannot efficiently find MEMs because a standard backward search is only unidirectional. Bannai et al. (2020) described a small auxiliary data structure for the *r*-index called thresholds that can be used to efficiently find MEMs. Thresholds consist of a set of *r*-length bit vectors that denote, for each subsequent pairs of runs of the same character, the position of the smallest value of the longest common prefix (LCP) array in the same interval. More intuitively, the thresholds’ data structure acts as a guide in the backward search, letting one know which value position to jump in the *r*-index once the range becomes empty. Bannai et al. (2020) did not describe how to construct the data structure in an efficient manner. This was accomplished later by Rossi et al. (2022), who demonstrated how to efficiently build the *r*-index along with thresholds.

### Prefix-free parsing

Although Gagie et al. (2018) showed that the *r*-index requires O(*r*) space to be stored, they did not show how to efficiently build the *r*-index. This problem was later resolved via the introduction of a preprocessing technique called *prefix-free parsing* (PFP), which creates both a parse and a dictionary of the input that is then used to construct the *r*-index (Boucher et al. 2019; Kuhnle et al. 2020). We briefly describe PFP as follows.

Given an input string *S* and two positive integers *w* and *p*, we append *w* copies of a delimiter symbol (say $) to *S* to create a cyclic representation, which we denote as *S*′, that is, *S*′ = $^*w*^*S*$^*w*^. For example, given the string GATTACAT#GATTAGAT#GATTACCAT, we create the circular string $$$GATTACAT#GATTAGAT#GATTACCAT$$$. Next, we use a Karp–Rabin (rolling) hash (Karp and Rabin 1987) to define the *trigger strings* (denoted as *E*) as the set of *w*-length strings whose hash value modulus *p* is equal to zero. Returning to our example, suppose *w* is equal to three and trigger string set *E* is equal to {$$$, TTA}; this leads to **$$$**GA**TTA**CAT#GA**TTA**GAT#GA**TTA**CCAT**$$$**, where the occurrences of trigger strings are shown in bold.

Once we identified the set *E* of trigger strings, we construct the dictionary *D* to be the set of all unique substrings that begin and end at a trigger string and contain no other trigger string. We sort the dictionary in lexicographical order. For our example, in which we have **$$$**GA**TTA**CAT#GA**TTA**GAT#GA**TTA**CCAT**$$$,** it follows that the dictionary *D* would be **$$$$$$**, **$$$**GA**TTA**, **TTA**CAT#GA**TTA**, **TTA**CCAT**$$$, TTA**GAT#GA**TTA**. Lastly, we define the parse *P* to be the representation of *S*′ via the ordering of the elements of *D*. Returning to our example, we have that *S*′ = 2,3,5,4,1. The efficient construction of the *r*-index from PFP is because it is constructed from *D* and *P alone*; the input string *S* is not needed for the construction of sampled SA and BWT. For the details of this construction, see Boucher et al. (2019) and Kuhnle et al. (2020).

### Index construction

Moni-align constructs the *r*-index (with the thresholds’ data structure) using the Moni build command with either (1) FASTA file(s) or (2) a reference FASTA file and a VCF file. The difference between building the index with input 1 or 2 is that the alignments will be lifted over to the linear reference if the index is built with input 2, but not with input 1. Moni-align will only index the variants present in the input haplotypes as represented in the VCF file. Moni-align is unable to construct the *r*-index from a graphical fragment assembly (GFA) file, as no algorithm currently exists for this task. The Moni build command runs several distinct steps in the background to build the index.

The build command first preprocesses the input using PFP. Using the PFP dictionary and parse, the build command constructs the thresholds’ data structure required for the *r*-index to efficiently find MEMs (Bannai et al. 2020; Rossi et al. 2022). Although the *r*-index, like the FM-index, performs exact pattern matching efficiently, it innately lacks an efficient method for partial matching, which is necessary for finding MEMs. Thresholds guide the *r*-index to continue a partial match when the backward search range becomes empty, indicating no perfect match between the read and any location in the reference genomes(s).

The build command then passes the PFP dictionary and parse to BigRePair (Gagie et al. 2019) for further grammar compression. This grammar is then passed to ShapedSLP (Gagie et al. 2020) to build random access data structures into the compressed genomes. For example, given an input string ATATGGGACAT and position *i*, these data structures can efficiently return the character at position *i* in a manner in which the input is compressed. Hence, this storage requires less space than storing the input in an array (or other uncompressed format) and is more efficient than accessing it on disk. We note that random access is needed to find the MEMs between the reads and the genome(s).

Lastly, during PFP construction, Moni-align generates the LevioSAM lifting files (Mun et al. 2021), which are needed to liftover the haplotype alignments back to the linear reference. The version of LevioSAM that is currently used in Moni-align needs a VCF file to construct the proper lifting files between the pangenome haplotypes and the linear reference genome, which is why alignment liftover is only supported with indexes built with a reference FASTA and VCF file.

### Alignment of reads

#### MEM-finding

Moni-align with the Moni align command can align both single-end and paired-end reads to the reference genome(s). The alignment process is almost identical for single-end and paired-end reads, but there are a few additional steps for paired-end alignment. The rest of this section goes through the paired-end alignment process.

The Moni align command initially estimates the insert size of the paired-end reads. By default, it finds the first 1000 reads that uniquely align to a location in the reference genome(s). It calculates different insertion size statistics such as mean (μ), standard deviation (σ), and variance for these reads using Welford's online algorithm (Welford 1962), an algorithm that requires only one pass through the data. Because insert size is inherently absent in single-end reads, Moni-align does not calculate these statistics for single-end data.

After estimating insert size (if applicable), the next step in alignment is finding MEMs between the read and the reference genome(s) in the forward and reverse directions. MEMs of any length can easily be found with the *r*-index using a simple two-pass algorithm that calculates matching statistics (Bannai et al. 2020). Given a read *R*[1…m] and sequence *S*, the matching statistics are an array *M*[1…m] such that *M*[i] contains the length of the longest prefix *R*[i…m] that occurs in *S*. In the backward pass, the positions in *S* of the longest prefix at each position in *R* are found using the *r*-index and thresholds. In the forward pass, the lengths of these prefixes are calculated using the SLP random access functionality, with a MEM being identified at the positions with nondecreasing lengths. By default, we keep MEMs of at least 25 bp and we additionally split MEMs >50 bp into two half-MEMs.

Considering all MEMs is ideal for the best alignments, but this approach quickly becomes impractical owing to high memory requirements and significant alignment slowdowns (Roberts et al. 2004). As a result, various filtering approaches are employed to reduce the MEM space while still finding high-quality alignments. A preprocessing MEM occurrence filter is implemented, which by default retains up to 1000 occurrences of a particular MEM per genome. We considered using a flat MEM occurrence filter, but this does not guarantee adequate MEM coverage across the genome(s) in the reference. A postprocessing MEM frequency filter is implemented, which by default removes MEMs that constitute 50% of the overall MEMs found for the read. This filter is based on the idea that frequently occurring MEMs are likely uninformative for read alignment. Another postprocessing filter implement is a MEM-orientation filter, which by default removes MEMs found on the strand that produces the smallest MEMs when the average size differs by ≥50 bp. Choosing an appropriate set of filters is hard, as it is highly dependent on the data. To help address this issue, an option is available to generate a CSV file containing relevant MEM statistics for each read.

#### Colinear chaining and full alignment

After finalizing the MEMs to keep, the MEMs are chained colinearly to find approximate alignments. We adopt the short-read chaining methodology and heuristics employed by minimap2 (Li 2018). Each chain has an associated score that indicates the quality of the alignment. By default, only chains that have a chain score of at least 40 are considered. Only the top five highest scoring chains are considered for full alignment by default. Full alignment involves performing base-level global alignment between the colinear MEMs of the chain using the KSW2 library (Li 2018; Suzuki and Kasahara 2018). Prior to doing full alignment, a heuristic is employed that checks the uniqueness of the chain. If the chain score and the lifted position of the left-most MEM of the chain match those of a previously processed chain, we assume the current chain is not unique and discard it. In rare cases, we might lose the best alignment to the pangenome, but we save significant time in practice with little accuracy reduction.

#### Orphan recovery

An orphan recovery feature is also implemented that attempts to rescue paired-end reads that have only one mate properly aligned. Using the aligned mate as an anchor, we attempt to find a suitable alignment for the other mate at a distance of μ ± 4σ. If a suitable location is identified, the paired alignment is reported. Otherwise, the properly aligned mate is treated as a single-end read and reported as unpaired.

#### Liftover

The last step in the alignment process is to lift the full alignments to the linear reference coordinates using LevioSAM (Mun et al. 2021). If two potential alignments of a read liftover to the same position, only the alignment with the higher score is retained. A BWA-like MAPQ score is calculated from the uniquely lifted-over alignments, and the best lifted-over alignment is reported in the SAM file. We note that the Moni-align alignments will not detect large structural variants, variants larger than the read, because there is no anchor in the reference to lift the alignments back to. The alignments are lifted to the linear reference genome so the SAM file can be used by existing downstream software, such as variant callers like DeepVariant (Poplin et al. 2018).

#### Mapping quality

The mapping qualities (MAPQs) computed by Moni-align are inspired by those calculated by BWA (Li and Durbin 2009; Li 2013). The single-end MAPQ values are computed as follows:

(1) $$Q=\min\left(60,\;6.02\;\times \;\frac{(s_{1}-s_{2})\;\times \;i^{4}}{m_{1}}-4.343\;\times \;\log(n+1)\right),$$

where *Q* is the single-end MAPQ value, *s*_1_ and *s*_2_ are the best and second-best single-end alignment scores among the lifted over alignments, *m*_1_ is the match score, *n* is the number of unique secondary alignment scores within a fixed range of the score of the best alignment, and *i* is a measure of confidence for the best alignment. The measure *i* is computed as follows:

(2) $$i=1-\;\frac{(m_{1}\;\times \;\ell )\;-\;s_{1}}{(m_{1}+m_{2})\;\times \;\ell },$$

where ℓ is the length of the read, and *m*_2_ is the mismatch score. Note that the MAPQ value increases as the dissimilarity between the best and second-best alignment (as well as other secondary alignments) increases. The paired-end MAPQ values for the first and second mates are calculated as follows:

(3) $$Q_{i}=\min\left(\max(Q,\min(Q^{\prime },\;Q+40)),\;6.02\;\times \;\frac{s_{1}-s_{2}}{m_{1}}\right),$$

where *Q*_*i*_ is the MAPQ of mate *i* (*i* ∈ {1, 2}), *Q* is the single-end MAPQ value of the mate, and *Q*′ is the paired-end MAPQ value. *Q*′ can be derived using Equation 1 with the exception that *s*_1_ and *s*_2_ represent paired alignment scores. The inner minimum and maximum functions of Equation 3 adjust the mapping quality of the individual mate MAPQ values based on the quality of the paired alignment. If the mapping quality of one mate in a pair is low and the other mate has a high mapping quality, the algorithm will elevate the low mate's MAPQ, reflecting the overall confidence in the paired alignment. The outer minimum function caps the MAPQ value assigned in cases in which it suspects the mate might originate from a tandem region.

### Software availability

The software for Moni-align is available at GitHub (https://github.com/maxrossi91/moni-align) under the GNU license and as Supplemental Code.

## Supplemental Material

Supplement 1

Supplement 2

Supplement 3
