# Supplementary material for: Accurate short-read alignment through r-index-based pangenome indexing
Source: Genome Res. 2025 Jul;35(7):1609–20. doi: 10.1101/gr.279858.124 (PMC12212348; doi:10.1101/gr.279858.124)
Supplement: Supplement 1 [file Supplemental_Experiment_Methods.pdf]

Supplement Experiment Methods

Constructing the pan-Chr 21 VCF files

We obtained the Chr 21 VCF files used to build the Chr 21 pangenomes by subsetting the full 1000 Genomes Chr 21 VCF file (http://ftp.ensembl.org/pub/data_files/homo_sapiens/GRCh38/variation_genotype/ALL.chr21_GRCh38.genotypes.20170504.vcf.gz) with the bcftools view command (v1.19) (Danecek et al. 2021). We created smaller Chr 21 VCF files that contained 10, 50, 100, 250, 500 haplotypes, with each consecutive larger VCF file being a superset of the previous file. We filtered variants such that only bi-allelic SNP and indel sites were retained.

Constructing the pan-Chr 21 FASTA files

The Chr 21 FASTA pangenomes for Bowtie 2, BWA, and minimap2 were generated using the bcftools consensus command (v1.19) (Danecek et al. 2021), using the GRCh38 Chr 21 FASTA file (https://www.ncbi.nlm.nih.gov/datasets/genome/GCF_000001405.40/) and the VCF files described in the Constructing the pan-Chr 21 VCF section. The consensus FASTA files included the same variants as the VCF files, ensuring consistency in genome representation.

Constructing the pan-MHC VCF file

The pangenome of the MHC region was constructed using the maternal and paternal haplotype-resolved assembly data from the 47 diverse individuals present in the HPRC Year 1 Version 2 data freeze (https://human-pangenomics.s3.amazonaws.com/index.html), in addition to T2T-CHM13 (https://github.com/marbl/CHM13), and GRCh38 (https://www.ncbi.nlm.nih.gov/datasets/genome/GCF_000001405.40/). Similar to other papers, we defined the MHC region on GRCh38 to span Chr 6:28510128-33480000 (Huijse et al. 2023). To construct the pan-MHC VCF file, the full HPRC assemblies were aligned to the GRCh38 reference with wfmash (v0.13.0) (Guarracino et al. 2024), producing a PAF file of the alignments. The PAF file was then processed with impg (v0.2.0) (Garrison et al. 2024b) to create a BED file that contained the assembly ranges corresponding to the GRCh38 MHC region. Ranges in the BED file that were within 100k bp of each other were merged using the bedtools merge command (v2.30.0) (Quinlan and Hall 2010). We extracted the corresponding BED ranges from the HPRC assemblies with the samtools faidx command (v1.19.2) (Li et al. 2009) and then ran pggb (v0.6.0) (Garrison et al. 2024a) on the extracted assemblies, producing a GFA file of the region. Next, we passed the GFA file to the vg deconstruct command (v1.55.0) (Garrison et al. 2018; Liao et al. 2023) to create the VCF file of the region, specifying the variants to be called in reference to GRCh38. Using shell scripting, we adjusted the positions of the variants in the VCF file to reference the start of

Chr 6 rather than the start of the MHC region. Lastly, with the bcftools view command, we filtered variants such that only bi-allelic SNP and indel sites remained and then normalized the variants with the bcftools norm command (v1.19) (Danecek et al. 2021).

Commands

Linear reference index

Moni-align: moni build -f <path to FASTA> -o <path to index> --tmp-dir <path to temp directory> -t 32

vg map: vg autoindex --workflow map --prefix <path to index> --ref-fasta <path to FASTA> --tmp-dir <path to temp directory> -t 32

vg giraffe: vg autoindex --workflow giraffe --prefix <path to index> --ref-fasta <path to FASTA> -t 32

HISAT2: hisat2-build -p 32 <path to FASTA> <path to index>

Bowtie 2: bowtie2-build --threads 32 -f <path to FASTA> <path to index>

BWA: bwa index -a bwtsw -p <path to index> <path to FASTA>

minimap2: minimap2 -t 32 -d <path to index> <path to FASTA>

Pangenome reference index

Moni-align: moni build -r <path to FASTA> -o <path to index> -v <path to VCF> --tmp-dir <path to temp directory> -t 32 -H 12 -S <path to sample list>

vg map: vg autoindex --workflow map --prefix <path to index> --ref-fasta <path to FASTA> --vcf <path to VCF> --tmp-dir <path to temp directory> -t 32

vg giraffe: vg autoindex --workflow giraffe --prefix <path to index> --ref-fasta <path to FASTA> --vcf <path to VCF> --tmp-dir <path to temp directory> -t 32

HISAT2: hisat2_extract_snps_haplotypes_VCF.py --non-rs <path to FASTA> <path to VCF> <path to SNP file>

HISAT2: hisat2-build -p 32 --snp <path to SNP file> <path to FASTA> <path to index>

Bowtie 2: bowtie2-build --threads 32 -f <path to FASTA> <path to index>

BWA: bwa index -a bwtsw -p <path to index> <path to FASTA>

minimap2: minimap2 -t 32 -d <path to index> <path to FASTA>

Alignment

Moni-align (MHC): moni align -i <path to index> -o <path to SAM> -1 <path to mate 1> -2 <path to mate 2> -t 32 --log-file <path to log> -w 10 -v 5 -f -d -S 1000 -u -a

Moni-align (Chr 21): moni align -i <path to index> -o <path to SAM> -1 <path to mate 1> -2 <path to mate 2> -t 32 --log-file <path to log> -w 10 -v 5 -f -d -S 2000

vg map: vg map -t 32 -d <path to index> -f <path to mate 1> -f <path to mate 2> --surject-to sam > <path to SAM>

vg giraffe: vg giraffe -t 32 -Z <path to GBZ> -m <path to MIN> -d <path to DIST> -f <path to mate 1> -f <path to mate 2> -o SAM > <path to SAM>

HISAT2: hisat2 -p 32 -x <path to index> -1 {path to mate 1} -2 <path to mate 2> -S <path to SAM>

GraphAligner: GraphAligner -t 32 -g <path to vg graph> -f <path to mate 1> <path to mate 2> -a <path to GAM> -x vg

Bowtie 2: bowtie2 --threads 32 -x <path to index> -1 <path to mate 1> -2 <path to mate 2> -S <path to SAM>

BWA: bwa mem -t 32 <path to index> <path to mate 1> <path to mate 2> <path to SAM>

minimap2: minimap2 -t 32 -ax sr <path to index> <path to mate 1> <path to mate 2> <path to SAM>

References

Danecek P, Bonfield JK, Liddle J, Marshall J, Ohan V, Pollard MO, Whitwham A, Keane T, McCarthy SA, Davies RM, et al. 2021. Twelve years of SAMtools and BCFtools. *GigaScience* **10**: giab008.

Garrison E, Guarracino A, Heumos S, Villani F, Bao Z, Tattini L, Hagmann J, Vorbrugg S, Marco-Sola S, Kubica C, et al. 2024a. Building pangenome graphs. *Nat Methods* **21**: 2008–2012.

Garrison E, Guarracino A, Kille B. 2024b. impg: implicit pangenome graph. https://github.com/pangenome/impg.

Garrison E, Sirén J, Novak AM, Hickey G, Eizenga JM, Dawson ET, Jones W, Garg S, Markello C, Lin MF, et al. 2018. Variation graph toolkit improves read mapping by representing genetic variation in the reference. *Nat Biotechnol* **36**: 875–879.

Guarracino A, Mwaniki N, Marco-Sola S, Garrison E. 2024. wfmash: whole-chromosome pairwise alignment using the hierarchical wavefront algorithm. https://zenodo.org/doi/10.5281/zenodo.10864529 (Accessed April 6, 2025).

Huijse L, Adams SM, Burton JN, David JK, Julian RS, Meshulam-Simon G, Mickalide H, Tafesse BD, Calonga-Solís V, Wolf IR, et al. 2023. *A pan-MHC*

reference graph with 246 fully contiguous phased sequences. Genomics http://biorxiv.org/lookup/doi/10.1101/2023.09.01.555813 (Accessed March 8, 2024).

Li H, Handsaker B, Wysoker A, Fennell T, Ruan J, Homer N, Marth G, Abecasis G, Durbin R, 1000 Genome Project Data Processing Subgroup. 2009. The Sequence Alignment/Map format and SAMtools. *Bioinformatics* **25**: 2078–2079.

Liao W-W, Asri M, Ebler J, Doerr D, Haukness M, Hickey G, Lu S, Lucas JK, Monlong J, Abel HJ, et al. 2023. A draft human pangenome reference. *Nature* **617**: 312–324.

Quinlan AR, Hall IM. 2010. BEDTools: a flexible suite of utilities for comparing genomic features. *Bioinformatics* **26**: 841–842.
