# Supplementary material for: Accurate short-read alignment through r-index-based pangenome indexing
Source: Genome Res. 2025 Jul;35(7):1609–20. doi: 10.1101/gr.279858.124 (PMC12212348; doi:10.1101/gr.279858.124)
Supplement: Supplement 3 [file Supplemental_Figure_S1.pdf]

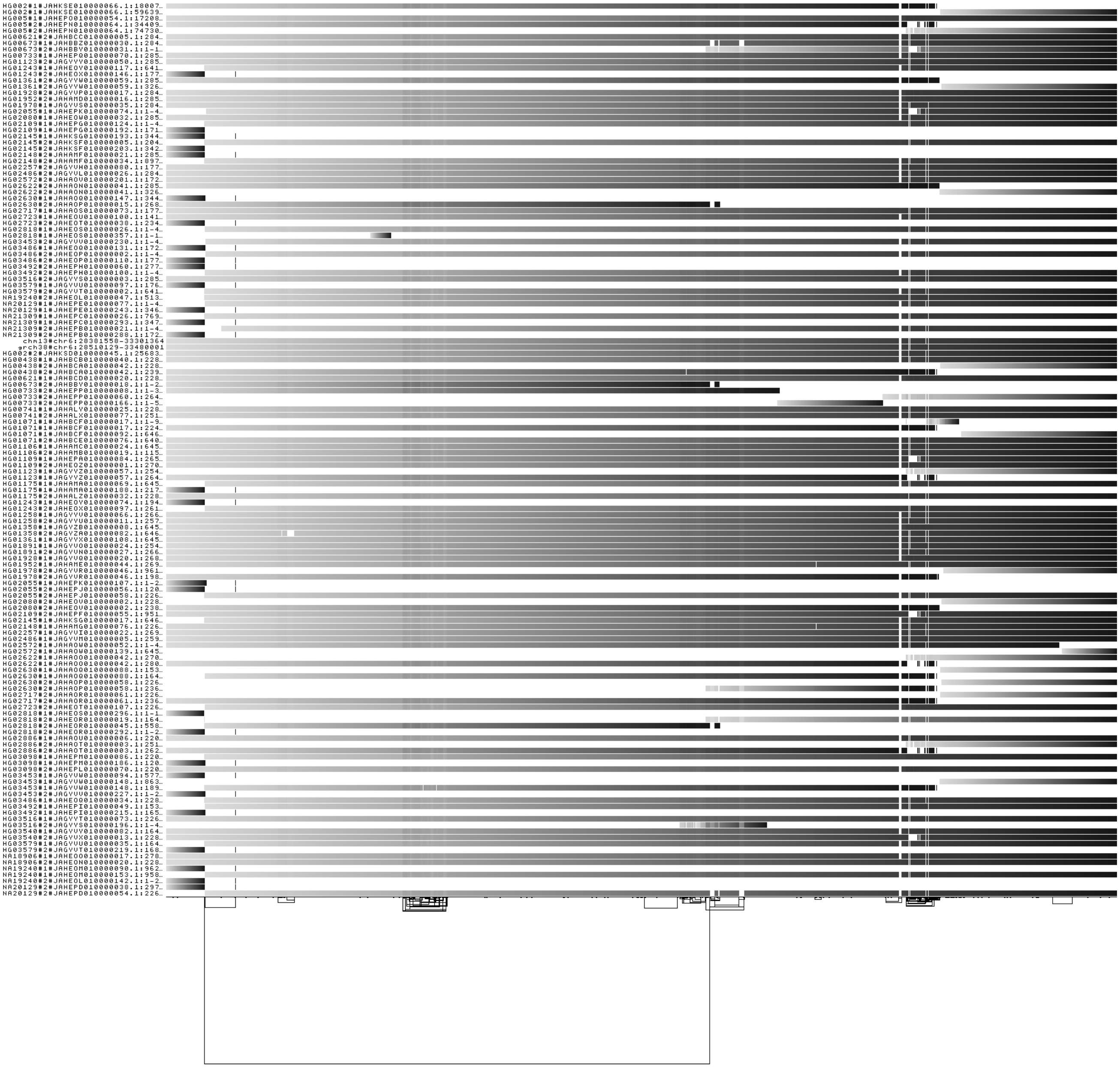

Supplemental Figure S1: odgi visual of the MHC region constructed. One of the plots produced by the odgi viz command by running pggb on the extracted MHC haplotypes of the HPRC assemblies. The plot colors the haplotypes by path position, with the color gradient transitioning from light to dark. Light colors represent the start of the sequence, and dark colors represent the end of the sequence. Many of the haplotypes go from left to right.
